# An Approach to Develop Digital Twins in Industry

**DOI:** 10.3390/s24030998

**Published:** 2024-02-03

**Authors:** Raúl González-Herbón, Guzmán González-Mateos, José R. Rodríguez-Ossorio, Manuel Domínguez, Serafín Alonso, Juan J. Fuertes

**Affiliations:** Grupo de Investigación en Supervisión, Control y Automatización de Procesos Industriales (SUPPRESS), Escuela de Ingenierías Industrial, Informática y Aeroespacial, Universidad de León, Campus de Vegazana s/n, 24007 León, Spain; rgonzh@unileon.es (R.G.-H.); ggonzm@unileon.es (G.G.-M.); jrodro@unileon.es (J.R.R.-O.); manuel.dominguez@unileon.es (M.D.); saloc@unileon.es (S.A.)

**Keywords:** digital twin, Industry 4.0, augmented reality, virtual reality, robotic electro-pneumatic cell

## Abstract

The industry is currently undergoing a digital revolution driven by the integration of several enabling technologies. These include automation, robotics, cloud computing, industrial cybersecurity, systems integration, digital twins, etc. Of particular note is the increasing use of digital twins, which offer significant added value by providing realistic and fully functional process simulations. This paper proposes an approach for developing digital twins in industrial environments. The novelty lies in not only focusing on obtaining the model of the industrial system and integrating virtual reality and/or augmented reality but also in emphasizing the importance of incorporating other enabled technologies of Industry 4.0, such as system integration, connectivity with standard and specific industrial protocols, cloud services, or new industrial automation systems, to enhance the capabilities of the digital twin. Furthermore, a proposal of the software tools that can be used to achieve this incorporation is made. Unity is chosen as the real-time 3D development tool for its cross-platform capability and streamlined industrial system modeling. The integration of augmented reality is facilitated by the Vuforia SDK. Node-RED is selected as the system integration option, and communications are carried out with MQTT protocol. Finally, cloud-based services are recommended for effective data storage and processing. Furthermore, this approach has been used to develop a digital twin of a robotic electro-pneumatic cell.

## 1. Introduction

Industrial digitization is taking place through the integration of various technologies into production processes, such as cloud computing, cyber–physical systems (CPS), cybersecurity, the Internet of Things (IoT), and digital twins [1]. These enabling technologies have brought about significant changes and emerging trends in the industry, marking a profound transformation known as Industry 4.0 [2]. This transformation has been proven to be a valuable asset for businesses, minimizing downtime and optimizing resource utilization, enhancing the overall efficiency of smart factories [3].

Digital twins are among the most interesting enabling technologies, capturing significant attention [4]. They are virtual representations of physical systems, staying synchronized with the real world at defined intervals and with precise accuracy [5]. This technology has the ability to predict responses to unexpected events through a bidirectional data exchange with the physical system and the utilization of algorithms that characterize the system’s behavior. Consequently, digital twins are highly advantageous for optimizing industrial processes, identifying failures and enhancing energy efficiency, among various other applications.

Creating a digital twin involves the development of virtual models that mirror the functional attributes of the physical system. In industry, these models have been usually designed employing specialized tools offered by automation system manufacturers [6]. However, graphics engines, traditionally employed in video game development, are becoming an alternative because of their ability to simulate complex scenarios and lower economic costs. They are cost-effective alternatives to the proprietary tools offered by automation system manufacturers. This paper proposes a methodology for the implementation of digital twins using one of these cost-effective alternatives, offering an analysis of the requirements and functionalities to be achieved. Furthermore, this methodology has been used to develop a digital twin for a robotic electro-pneumatic cell.

The main contribution of this work is a method for developing digital twins that provides a realistic industrial environment where several enabling technologies of Industry 4.0 are seamlessly integrated to interact with real equipment. These technologies include automation, robotics, connectivity with standard protocols, system integration, cloud-based storage and data processing, machine learning, virtual and augmented reality, among others. Most of the previous works are centered on obtaining industrial system models and incorporating virtual and/or augmented reality to enhance the functionality of digital twins. However, they do not focus on the importance of adding other enabled technologies of Industry 4.0 to the industrial digital twin. It is crucial to assess the integration of complementary technologies to develop a digital twin that supports real-time simulation and analysis. This integration facilitates optimization, predictive maintenance and enhanced monitoring, ultimately leading to improved operational efficiency. The proposed methodology aims to achieve the following objectives:

- To develop precise scenarios of virtual and augmented reality. There are open-source graphic engines that, although initially associated with video game development, are progressively being utilized for engineering, marketing, and architectural applications. Additionally, these engines are equipped with a powerful physics engine that streamlines the 3D modeling of industrial systems and enables the adaptation of applications to augmented reality.
- To manage automation and robotic systems based on distributed and decentralized architectures where control systems communicate with each other through standard industrial communication protocols, aiming to enhance efficiency, speed, and repeatability.
- To ensure connectivity using standard communication protocols, such as Message Queuing Telemetry Transport (MQTT), and specific industrial protocols that are traditionally used for the operation or configuration of control systems, such as PROFINET, Modbus TCP, etc. Also connectivity with OPC UA, which is a modern industrial communication standard, is based on the classic client/server architecture.
- To achieve system integration, with tools such as Node-RED, an open-source visualization tool created by the IBM Emerging Technology team that allows for interconnecting all the elements of the Internet of Things.
- To store and process data in the cloud. Virtual machines or services allocated in the cloud have advantages over other local tools, including cost-effectiveness, flexibility, continuous support, and heightened security. Furthermore, cloud services enable the implementation of data analysis techniques by facilitating real-time and decentralized decision making.
- To add industrial cybersecurity. Although industrial cybersecurity, as well as other main enabling technologies in digital industry, have not been incorporated into our functional digital twin proposal, the robotic electro-pneumatic cell used for experimentation currently features an industrial firewall to ensure network security. The plan for incorporating this aspect into the digital twin in future work is described.

This paper is organized as follows: Section 2 provides a current state of the art with an overview of the technologies employed in digital twin development; next, Section 3 explains the proposed methodology to develop a digital twin. Section 4 presents the digital twin developed for a robotic electro-pneumatic cell using the proposed methodology. The conclusions of the paper are in Section 5.

## 2. Digital Twins

The digital twin is not a novel concept but rather integrates other previously recognized components such as 3D modeling, simulations, or Product Life-cycle Management (PLM) [7,8]. This concept can be described as the utilization of data-driven insights for the supervision, administration, and ongoing enhancement of a system or product [9]. However, the actual trend of fusing the physical world with the virtual world has significantly increased the popularity of this concept [10], following its introduction by Grieves through its relation to Product Life-cycle Management and the concept of “Mirrored Spaces Models” [11,12]. This trend has evolved from the concept presented by NASA and the U.S. Air Force in 2012 [13,14,15] to an expansion of the term presented by Grieves [16] and, subsequently, in collaboration with Vickers [17] defining three components (real space, virtual space and data link information process) as the basis for its development. In addition, the complexity of the systems is increasing, so the incorporation of other technologies such as augmented reality helps to work better with the digital twin data [18,19].

There are several previous works on digital twins for industrial applications in multiple sectors [20]. In [21], the authors present a digital twin that incorporates an anomaly detection system for asset monitoring, along with its data integration method based on extended IFCs (Industry Foundation Classes), in daily operation and maintenance management. In addition, they incorporate a case study applying anomaly detection on centrifugal pumps in HVAC (Heating, Ventilation, and Air Conditioning) systems. In [22], the authors propose a model for predictive maintenance of a CNC (Computer Numerical Control) machine tool using the data and the digital twin model. This model is used in a case study on cutting tool life prediction. Another example of predictive maintenance can be found in [23], where a methodology for calculating the Remaining Useful Life (RUL) of machinery equipment by utilizing physics-based simulation models and the digital twin concept is presented and applied to an industrial robot. In other sectors such as wind farms, the use of predictive digital twins is also interesting [24]. This paper presents a digital twin based on Unity for visualization, OPC-UA for communications and the Prophet prediction algorithm to detect potential failures in wind turbine components. In addition, the authors incorporate augmented reality to enhance user experience. In the automotive sector, digital twins for connected and automated vehicles (CAVs) also stand out [25]. In this work, a digital twin simulation architecture is proposed. This architecture is based on three layers: Unity game objects to represent the “CAVs hardware”, Unity scripting API to represent the “CAVs software” and external tools for the simulation functionalities. These works focus on acquiring the model of the industrial system and integrating virtual reality and/or augmented reality to improve functionalities of the digital twin. However, they do not define the importance of incorporating other enabled technologies of Industry 4.0 into the industrial digital twin. These technologies include system integration, connectivity with standard and specific industrial protocols and cloud services, or new industrial automation systems. One of the main contributions of this work is to propose a method to develop digital twins where these enabling technologies are integrated to interact with the real system and work collectively. Furthermore, a proposal of the software tools that can be used for this is made.

Currently, there are many tools on the market for the development of digital twins [8]: Siemens has the NX family of software that, together with SIMIT and PLCSIM Advanced, form a pack to develop digital twins; Rockwell Automation provides a software called Emulated 3D for this purpose. These tools have the disadvantages of not being very flexible in their tasks and of being completely dependent on the manufacturers as they are proprietary solutions. There are also cloud alternatives as offered by Amazon with AWS IoT TwinMaker or Microsoft with Azure Digital Twins. Alternatively, there are free open-source tools such as Easy Java/JavaScript Simulations (EJS), Unreal Engine or Unity. This tools have the advantage of not depending on any manufacturer by having a very strong support community behind them. In addition, the access to the source code allows a great flexibility in the development. EJS stands as a versatile development and modeling tool, facilitating the construction of Java and JavaScript applications through straightforward programming. There are widespread applications in the developing of simulations and remote laboratories [26,27]. This is due to the possibility of establishing communication with physical systems over the network, its capacity to model using physical equations, and its user-friendly implementation of applications accessible through web browsers and mobile devices. On the other hand, tools like Unreal Engine or Unity provide an extra benefit compared to the previously mentioned ones, as they are equipped with a physics engine that enhances the 3D modeling of industrial systems. Furthermore, these tools are cross-platform, easing the adaptation of applications to augmented reality and virtual reality environments [28]. Unity and Unreal Engine are open-source graphic engines primarily associated with video game development. However, they are progressively being used together with frameworks for engineering, marketing, and architectural applications [29].

Unity serves as a real-time 2D and 3D application development platform, offering a multitude of benefits. Featuring object-oriented programming in the C# language, it proves to be a versatile tool applicable to various sectors: video games, engineering, construction and architecture. In particular, it is multi-platform compatible, enabling the export of applications developed for virtual reality (VR) and augmented reality (AR) to platforms such as Windows, Linux, Android, iOS, or even web pages. Furthermore, since it is programmed in C#, it provides support for a wide range of communication protocols as Modbus TCP, OPC UA, HTTPS, MQTT, and others. Unity easily integrates with other widely used tools like Blender and Maya, and it seamlessly interacts with other digital twin development platforms such as Azure Digital Twins by Azure and SIMIT by Siemens. Finally, it benefits from an active community and comprehensive documentation, offering substantial resources for 3D application development.

## 3. Methodology to Develop Digital Twins

**Figure 1:**
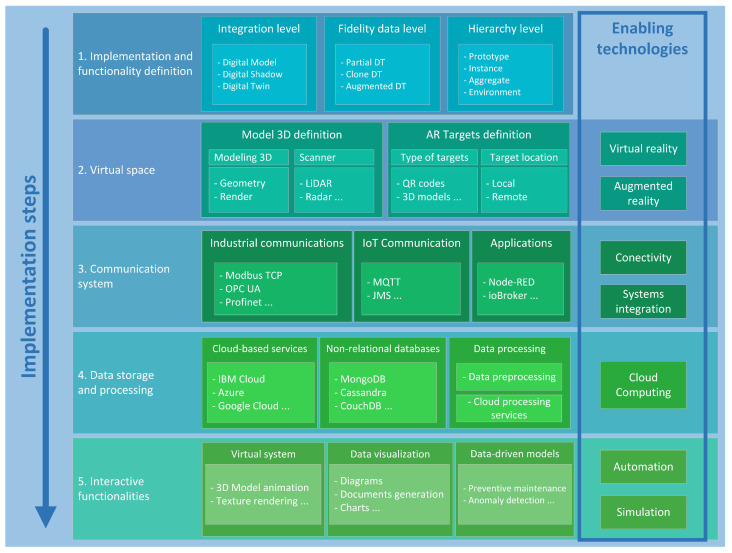
Methodology to develop digital twins.

Implementing digital twins requires a methodical approach to ensure an efficient development. As previously discussed throughout the document, the main aim of this work is to propose a method to develop digital twins that provides a realistic industrial environment where the multiple enabling technologies of Industry 4.0 are integrated to interact with real equipment and to work collectively. Below, we outline a set of guidelines describing the key steps involved in creating digital twins and linking them to enabling technologies. This proposed method is graphically represented in Figure 1.

### 3.1. Implementation and Functionality Definition

Initially, it is fundamental to define the intended functionality of the model, whose considerations will form the basis for development. This involves defining the level of detail desired. To achieve this, a decision must be made regarding how to establish communication between the application and the real system. If there is no bidirectional data communication where the changes between the system and the application are not reflected, it will be considered as a digital model. If changes are reflected in both directions, it will be classified as a digital twin. However, if changes are only reflected from the physical system to the application, it will be a digital shadow [5,30]. In addition, it is also necessary to define the fidelity of the data to be used, implying the usage of only some relevant variables from the system (Partial DT), all the relevant variables (Clone DT) or all the possible variables and also historical data (Augmented DT). Furthermore, it is necessary to decide the purpose for which the digital twin application is to be used with respect to the hierarchy of the system: if it is employed to make decisions regarding the design of the future process, it is called Prototype DT; if, in contrast, it is used as a digital model of a specific system element, it is named Instance DT; if it is a set of system elements, it is defined as Aggregate DT, and if it is a set of Aggregate DTs, it forms what is known as Augmented DT [7,17]. Finally, other considerations, such as the implementation of augmented reality functionalities, should be taken into account in this step.

### 3.2. Virtual Space

This step involves acquiring a three-dimensional model of the real system, according to the predefined specifications. Various methods can be used: CAD/CAE design tools, such as CATIA, SolidWorks etc., software-based scanners that leverage the device’s cameras and sensors (e.g., LIDAR sensors in iOS), or professional scanners [8]. Next, this 3D model has to be imported into a real-time 3D development platform, like Unity software. If augmented reality wants to be integrated, Unity offers various methods. The recommended one for this purpose is using a SDK (Software Development Kit known as Vuforia. Developed by PTC, Vuforia seamlessly integrates with Unity [31], using computer vision to identify elements referred to as targets. Once these targets are detected, Vuforia triggers an event that Unity captures, simplifying the development of the AR application. Consequently, targets are defined as the elements that the application must recognize to enable interaction with the real system. Targets can take the form of QR codes linked to specific elements or components of the system, or they may be 3D models representing parts of the system for recognition. Furthermore, a decision must be made regarding the storage of these targets: they can either be stored locally within the application itself or hosted remotely, such as in a cloud-based repository.

### 3.3. Communication System

This step involves establishing a communication system that enables bidirectional interaction between the digital model and the physical system. Communication links, with a wide range of devices and applications, have to be defined [32]. For this, Message Queuing Telemetry Transport (MQTT) [33] communication protocol must be highlighted. It is a publisher/subscriber approach where a central element, known as the ‘broker’, is in charge of directing all data generated under a specific ‘topic’ (essentially, a variable tag) to subscribed clients. This approach eliminates the need for listening confirmation and simplifies the integration of new devices. MQTT shares this messaging pattern with other protocols such as Java Messaging Service (JMS), Extensible Messaging and Presence Protocol (XMPP) or Advanced Message Queuing Protocol (AMQP) [34]. Another option is to use protocols like Modbus TCP, Profinet, etc. or the OPC UA standard, which is designed for industrial communications. OPC UA uses the same publisher/subscriber mechanism and incorporates multiple security measures [35]. To manage all these communications of different nature, a variety of interconnection tools have emerged, which are mainly rooted in visual programming environments. In these tools, the organization of data connections is represented through flows, each one composed of several nodes that serve specific functions, similar to the functions found in text-based programming languages [36]. Among the available tools, it is noteworthy to mention Node-RED, which has gained popularity due to its strong and engaged community support, its extensive utilization across diverse application domains, and its growing adoption for the development of digital twins [37]. There are additional tools available for similar purposes: Crosser [38], known for its integration with Python and its machine learning capabilities; ioBroker [39], primarily geared toward home automation; and OPC Router [40], with a focus on standardizing information exchange within the industrial domain.

### 3.4. Data Storage and Processing

It is necessary to select a data storage system that facilitates the collection and utilization of large amounts of data by the digital twin. Ensuring fast and efficient access to these data can be a complex task, as approaches focused on local storage and processing are suitable only for smaller systems. The Internet of Things (IoT), accompanied by the proliferation of devices generating and consuming different data types, also implies the need for data processing methods capable of addressing the decentralized nature of the information. In this regard, several options have emerged for the development of cloud-based services, including IBM Cloud, Microsoft Azure, Amazon Web Services, and Google Cloud [41]. Alternatively, it is possible to select on-premise non-relational databases like MongoDB, CouchDB, Cassandra, and Neo4J [42,43]. Furthermore, for complex systems, implementing data processing and data preprocessing using cloud computing techniques lightens the computational load of the digital twin.

### 3.5. Interactive Functionalities

After establishing the communication and data management system, the next phase is to use these data to develop the digital model with the functionalities defined in the first phase. Animating various components of the system, energizing mobile parts, or creating visual representations by using different colors to mirror real-time behavior might take place in this phase. Furthermore, it is valuable to visualize these data in a similar way to a monitoring system. This can involve employing various graphs or diagrams or generating CSV documents that facilitate an in-depth analysis of the acquired information. In addition, interactive functionalities associated with data-based models can be incorporated for preventive maintenance, anomaly detection, the simulation of different operating states, etc.

**Figure 2:**
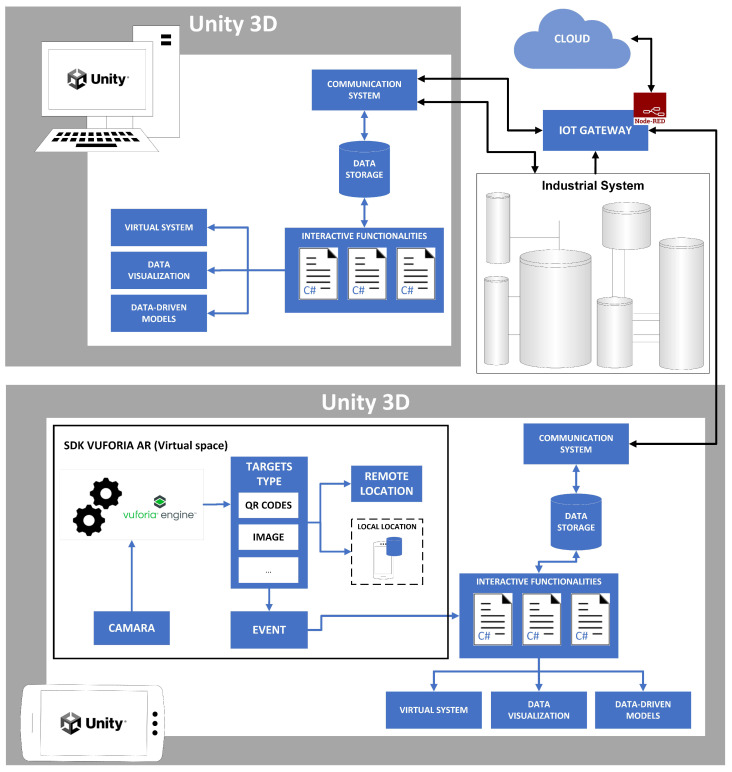
Unity applications scheme.

Based on the proposed method, a schema for developing a digital twin with direct bidirectional communication with the industrial system is shown in Figure 2. This proposal has been used to develop a digital twin for a robotic electro-pneumatic cell, as explained in the next section. To define the virtual space, it is necessary to obtain a 3D model of the system using some of the options presented previously. A VR application (computer application), at the top of the figure, and an AR application (mobile device application), at the bottom, are developed with Unity. Interactive functionalities such as animations, texture rendering, data visualization, etc., have to be defined for each of the applications. The AR application is based on the Vuforia SDK that collects the necessary information through the mobile device’s camera and uses computer vision techniques to identify targets. Once these are identified, an event is triggered in the application to perform the interactive functionalities. The Node-RED system integration tool is running on an IoT gateway. It uses services provided by the cloud for deploying cloud computing applications and decentralized storage. Node-RED can also use edge computing methods to preprocess system data. Finally, industrial communications such as Modbus TCP and/or OPC UA are used to receive data from the industrial system and the MQTT protocol to send data to the cloud.

## 4. Results

**Figure 3:**
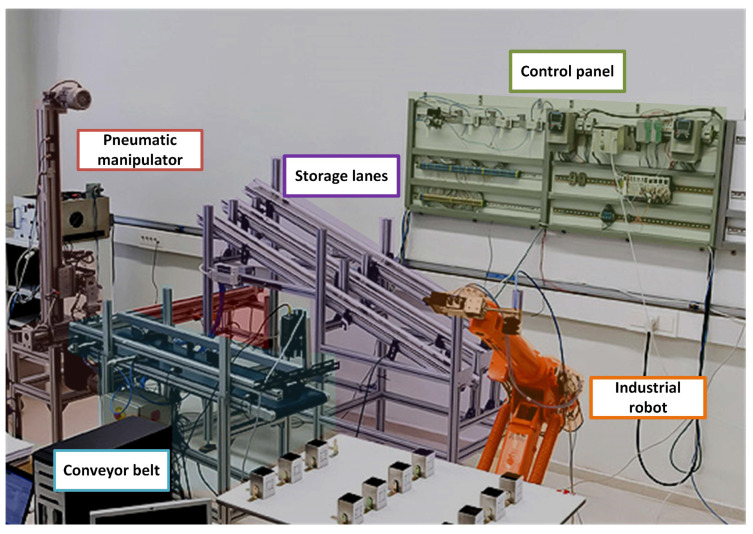
Robotic electro-pneumatic cell.

The proposed method has been employed to create a digital twin for a robotic electro-pneumatic cell. The cell performs the classification of pieces, emulating a production process (see Figure 3). The first subsystem of the cell is a conveyor belt equipped with pneumatic cylinders. This belt uses photoelectric sensors to detect the presence of pieces and a video camera for identifying them through an alphabetical code. There are six cylinders, three on the left of the belt (L1, L2, L3) and three on the right (R1, R2, R3). Three different sequences for the pneumatic cylinders can be selected for each processed piece to simulate different production processes. Sequence 0 is (R1-L1-L2-R3), sequence 1 is (R2-L1-L2-R3), and sequence 2 is (L1-L2-R3).

The second subsystem is the pneumatic manipulator, which is situated at the end of the conveyor. Its function is to collect the pieces and redirect them to the three storage lanes. The pieces are classified in these lanes based on the codes printed on them, which is a task performed by the video camera in the first subsystem. The last subsystem is an industrial robot that loads the pieces at the conveyor belt when each cycle starts and retrieves the pieces stored in the lanes when the cycle finishes. All the aforementioned subsystems are controlled using up-to-date controllers that support distributed architectures and programming paradigms. This enables not only high processing capabilities but also event-driven control strategies, which are more convenient within the current industrial reality. The distributed architecture includes two frequency inverters whose aim is to control motors associated with the conveyor belt and the manipulator. Communication with these inverters is achieved using Modbus TCP protocol. On the other hand, the industrial robot operates independently, equipped with its own control system and strategy, but in order to facilitate information exchange, multiple signals are wired from an I/O module to the external signal card of the robot. Leveraging these signals, a controller shares information and triggers various robot movements based on the cell’s state. Even though these up-to-date controllers also offer superior memory protection functionalities and options to prevent program or subsection modifications, the system is provided with an industrial firewall in order to ensure a series of filtering rules to apply to the traffic, restricting communication with the control devices. The operation of the electro-pneumatic cell and a web version of the developed application can be explored on the research group’s website (SUPPRESS website (accessed on 2 January 2024): https://lra.unileon.es/physical-systems/robotic-electropneumatic-cell/simulations/).

**Figure 4:**
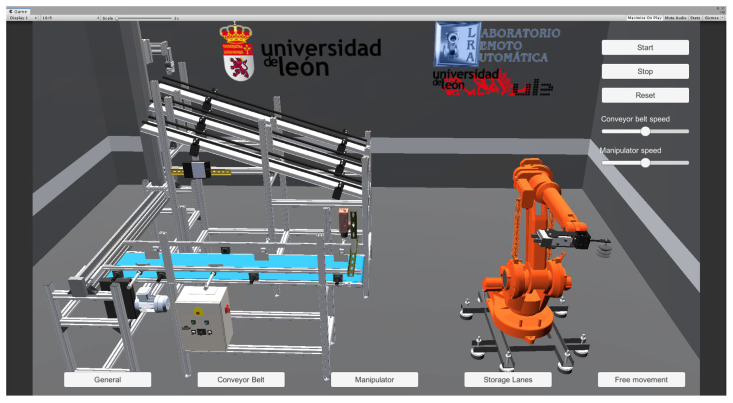
Virtual reality application with Unity.

The VR application of the robotic electro-pneumatic cell is shown in Figure 4. The real-time 3D development platform used for its creation is Unity. Although originally linked to video game development, Unity is increasingly finding applications in engineering, marketing, and architecture. This trend is attributed to its robust physics engine and its seamless adaptability to augmented reality. The 3D model of the system was created using the well-known 3D CAD design software SolidWorks. The VR application receives real-time operation data from the robotic electro-pneumatic cell through Modbus TCP protocol and utilizes this information to animate various parts of the 3D model, including the manipulator, conveyor belt, and other components. The VR application features a manual operation mode allowing adjustments to system parameters, including the speed of internal elements, piece selection for sorting, unrestricted free movement within the workspace, etc. In automatic operation mode, pieces are classified based on the codes printed on them. In addition, the application provides an option to generate a CSV file for storing the generated information.

The IoT gateway used is a Harmony IIOT EDGE Box from the manufacturer Schneider Electric. The Node-RED system integration tool is running on it. This tool reads system data via the Modbus TCP protocol and sends it to the IBM Cloud, which is the chosen data storage and processing system, using the MQTT standard. The VR application also communicates with the IoT Gateway using the Modbus TCP protocol to obtain data coming from the cloud. These devices and technologies guarantee connectivity by employing established IoT communication protocols and industry-specific protocols typically utilized for controlling or configuring systems.

**Figure 5:**
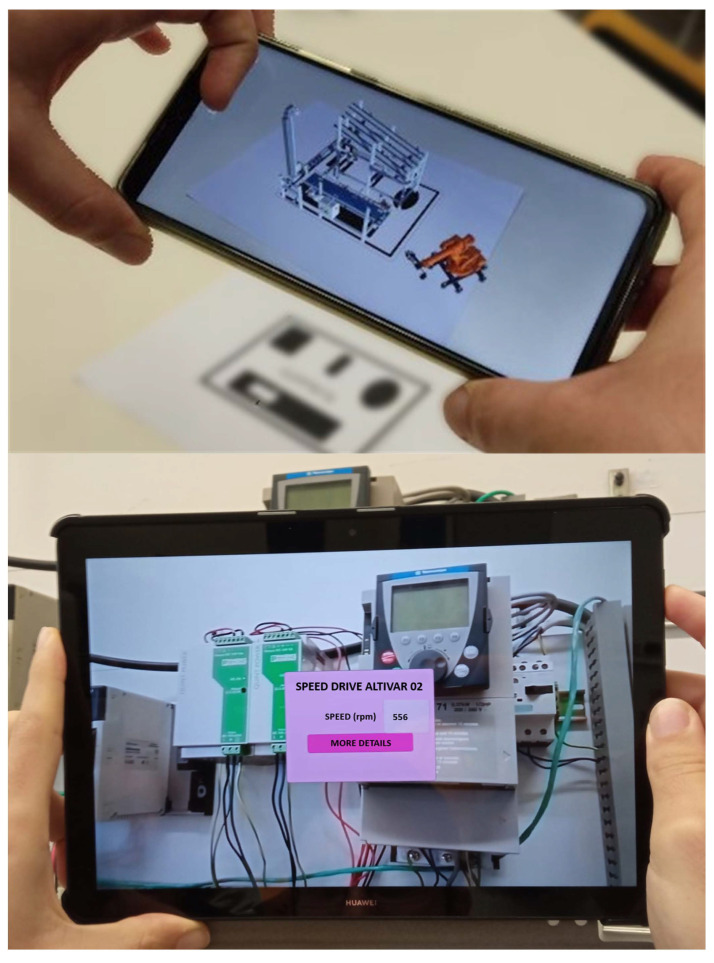
Augmented reality application.

The developed digital twin has augmented reality capabilities through a mobile application (see Figure 5). Vuforia in combination with Unity has been used to develop this AR application. It captures images using the device’s camera and overlays process-related information onto these images. This information is updated in real time and is sourced from the app’s communication with the IoT Gateway using Modbus TCP. The captured images (targets) have been developed using QR codes placed on the devices. These targets have been previously uploaded to the application for real-time recognition. At the bottom of the figure, an example of use with one of the variable speed drives (used for the conveyor belt and the pneumatic manipulator) can be seen, showing a box with the main operating parameters and a button to display more detailed information on the current status. At the top of the figure, another functionality can be seen, representing the general status of the system in a smartphone.

**Figure 6:**
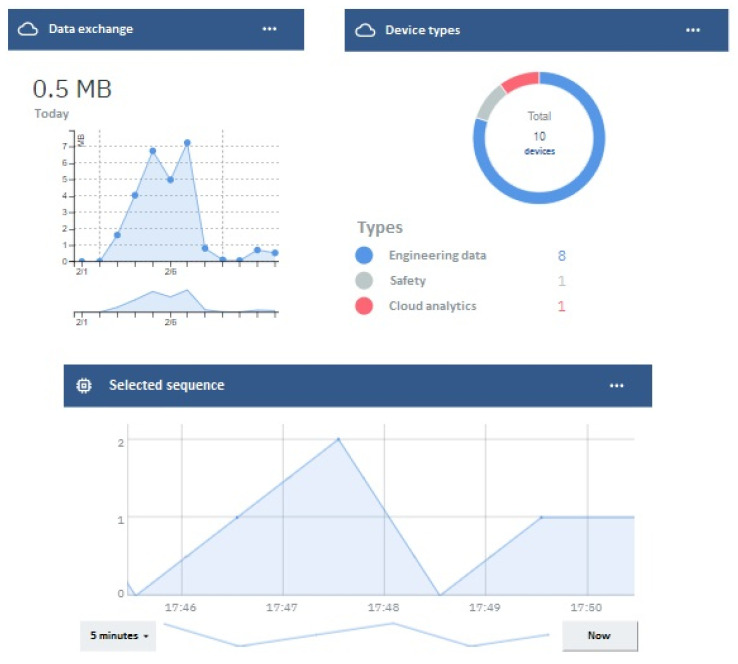
Digital twin dashboard in the cloud.

Finally, in Figure 6, a digital twin dashboard running in the IBM Cloud is shown. This dashboard provides information about communications with the cloud and the current cylinder sequence. At the bottom, the cylinder activation sequence over time is represented, showing the sequence as 0-1-2-0-1. In the top-left corner, data traffic in megabytes per day is displayed, while on the right side, the number of devices connected to the IoT Platform is shown. The IoT platform is an IBM Cloud service used to enable communication between the IoT Gateway and other cloud services. In the figure, it can be seen that ten different devices are connected at that moment. Every device represents a logical abstraction related to data with common characteristics. It can be a physical device (such as electrical variables read from an engine) or a set of data with the same nature (like signals associated with different security beacons). Eight physical devices have been created to rationalize and organize engineering data generated during the process, while another two devices have been defined separately with the aim of organizing data associated with safety considerations and cloud computing calculations. Each device has a specific token associated with it to secure communication with the IoT Platform. In this way, different nodes with the same token can be deployed to communicate various data units to the IoT Platform under the same device identification. These cloud-based services offer distinct advantages over local tools, presenting cost-effectiveness, flexibility, ongoing support, and enhanced security. Moreover, these services empower the implementation of data analysis techniques, facilitating real-time and decentralized decision making.

In summary, Table 1 provides a comparison between our digital twin solution and those proposed by the works discussed in Section 2. These works only present a partial implementation of the aforementioned enabling technologies. In contrast, our work aims to highlight the importance of the combined implementation of Industry 4.0-enabling technologies for the development of digital twins.

## 5. Conclusions

This paper presents a method for developing digital twins in industrial environments. Creating virtual spaces that include innovative features such as augmented reality provides a more interactive and immersive approach to monitoring and improving industrial process efficiency. The complexity of deploying a digital twin partly arises from the need to combine different technologies to facilitate the connection between devices of diverse nature. Additionally, it involves addressing the storage and processing of large volumes of data. Another challenge in the development of digital twins is their demands for fidelity and fast response times, which further complicates the difficult task of technological integration.

The first phase for creating digital twins involves a definition of the expected functionality of the model. This includes specifying the desired level of detail, the data communication integration or the fidelity of the system. The next step is to define the virtual space, which consists of acquiring the 3D model of the process using CAD/CAE software or a professional scanner. Then, this model must be imported into a real-time 3D development platform.Unity is the cross-platform choice for this approach because it is equipped with a physics engine that streamlines the 3D modeling of industrial systems. An essential consideration is the integration of augmented reality capabilities. Adapting Unity applications to augmented reality is easy with the Vuforia SDK. It uses computer vision to identify targets and trigger events captured by Unity, simplifying AR application development.

Next, it is necessary to define the communication system that facilitates two-way interaction between the digital model and the physical system. In this case, industrial communications such as Modbus TCP or OPC UA must be highlighted. These protocols allow easy communication between the VR/AR application and the industrial system. Another required communication for this implementation is the connection to the cloud. To this end, the MQTT communication protocol is of particular interest. This protocol eliminates the need to listen to confirmation and speeds up the integration of new devices. In addition, interconnection tools should be used, particularly those based on visual programming environments. Among these tools, Node-RED stands out for its widespread adoption, community support, and increasing utilization in various application domains. Finally, it is essential to choose a data storage system that can effectively handle the extensive data generated from a digital twin. The best option for ensuring rapid and efficient data access are cloud-based services, where also powerful data processing methods can be used.

The proposed methodology for developing digital twins offers several advantages. Firstly, it provides a more comprehensive understanding of the modeled process. Real-time interaction and the dynamic visualization of physical systems in virtual space enable engineers and operators to comprehend the complexity of processes and make informed decisions. This information leads to improved process control, faster problem resolution, and optimized overall efficiency. The proposed method also serves as a reference for similar approaches in the development of digital twins applied to other industrial processes. By creating a standardized framework, it becomes a useful resource for industry professionals looking to implement digital twin solutions in a variety of situations. It is important to note some limitations related to the current methodology, such as the impossibility of working with certain real-time constrains, because using cloud-based tools implies latency and dead times, which can be unacceptable in some specific environments. Another limitation could be the current impossibility of using the developed digital twin for advanced cybersecurity analysis, although improvements in this field have been conceptualised for future works.

Regarding future work, it is recommended to conduct further testing on other plants or industrial systems to verify the effectiveness and adaptability of the digital twin methodology in different environments. Additionally, it is important to ensure that the digital twin methodology can be extended to larger plants or processes, thus evaluating the performance and feasibility of the developed models when applied to industrial systems of high complexity and scale. The successful expansion paves the way for widespread adoption, making digital twins a versatile solution for a variety of industrial environments. On the other hand, some improvements in the field of cybersecurity should also be addressed, such as replacing the industrial firewall with a more advanced device such as an Intrusion Prevention System (IPS), which allows a thorough analysis of the network traffic in the plant. The implementation of such devices would enable the collection of information on the analyzed traffic to be incorporated into the digital twin. With these data, the digital twin could be enhanced with the ability to detect anomalous or even malicious traffic compared to normal traffic. Future research should address these issues to contribute to the maturity and widespread application of digital twin technology in industrial settings.

## Figures and Tables

**Table 1:** Comparison of different digital twin solutions.

Referenced Work	Enabling Technologies	
	**VR**	Software BIM
[21]	**Automation**	Centrifugal pumps
	**Connectivity**	Industry Foundation Classes (IFCs)
	**VR**	CAD/CAE software
[22]	**Automation**	CNC machine tool
	**Connectivity**	Mapping interface (OPC UA, Sockets, …)
	**Automation**	Six-axis robotic structure
[23]	**Connectivity**	MQTT
	**System integration**	Signal processing gateway
	**VR**	Unity
	**Automation**	Sensors for monitoring
[24]	**Connectivity**	OPC UA
	**System integration**	Node-RED
	**AR**	Vuforia Engine with Unity
	**VR**	Unity
	**Automation**	Connected and automated vehicles (CAVs)
[25]	**Connectivity**	MQTT and sockets
	**System integration**	Unity Scripting API
	**Cloud**	Computing with AWS
	**VR**	Unity
	**Automation**	Robot and distributed architecture
	**Connectivity**	Modbus TCP, OPC UA and MQTT
Current work	**System integration**	IoT Gateway with Node-RED
	**AR**	Vuforia Engine with Unity
	**Cloud**	Storage and computing with IBM Cloud
	**Cybersecurity**	Industrial firewall

## Data Availability

The raw data supporting the conclusions of this article will be made available by the authors on request.
